# Predictive value of the quantitative flow ratio after pretreatment and drug-coated balloon therapy for functional stenosis of target vessels at the mid-term follow-up

**DOI:** 10.3389/fcvm.2025.1610386

**Published:** 2025-09-25

**Authors:** Xinjun Lin, Yuwei Chen, Wanjun Huang, Zhendong Cheng, Bin Yu, Wei Ji, Yaoguo Wang, Chaoxiang Xu

**Affiliations:** ^1^ The Second Clinical College of Fujian Medical University, Quanzhou, Fujian, China; ^2^ Department of Cardiology, The Second Affiliated Hospital of Fujian Medical University, Quanzhou, Fujian, China; ^3^ Fujian Key Laboratory of Lung Stem Cell, Department of Pulmonary and Critical Care Medicine, The Second Affiliated Hospital of Fujian Medical University, Quanzhou, Fujian, China

**Keywords:** quantitative flow ratio, drug-coated balloon, pretreatment, functional stenosis, target vessel

## Abstract

**Background:**

Drug-coated balloon (DCB) treatment requires adequate preconditioning of target lesions. The quantitative flow ratio (QFR) is an emerging method for assessing functional stenosis of coronary arteries. This study investigated the predictive value of the QFR after pretreatment and DCB treatment for functional stenosis of the target vessel at the mid-term follow-up.

**Methods:**

The study included patients with coronary heart disease who received DCB treatment. The patients were divided into two groups based on their functional stenosis status at follow-up. Independent predictors associated with functional stenosis and the effects of the pretreatment QFR on outcomes were investigated. A receiver operating characteristic curve analysis was used to determine the pretreatment QFR cutoff value associated with follow-up QFR >0.80. The mediating effects of the QFR after pretreatment and DCB treatment on outcomes were also examined.

**Results:**

The study included 97 target vessels from 97 patients; the median follow-up time was 297.0 days. The high (QFR >0.80) and low (QFR ≤0.80) QFR groups included 78 and 19 vessels, respectively. Multifactor logistics regression analysis identified the pretreatment QFR as an independent predictor of outcome. The pretreatment QFR cutoff value for predicting functional stenosis was 0.705. The DCB-treatment QFR did not elicit mediating effects on the follow-up QFR.

**Conclusions:**

The QFR after pretreatment directly and significantly affected the follow-up QFR. Pretreatment QFR <0.705 may be a valuable predictor of functional stenosis after DCB treatment.

## 1 Introduction

Coronary artery disease (CAD) is a common cardiovascular disease that threatens human health. Percutaneous coronary intervention (PCI) remains the mainstay of treatment for CAD (1). Percutaneous transluminal coronary angioplasty and its derivative techniques, including drug-coated balloon (DCB) treatment, have become important components of PCI. Although drug-eluting stents (DES) have become a widespread CAD treatment, DCB treatment retains unique advantages. For instance, DCB treatment can inhibit intimal hyperplasia without leaving a permanent implant by coating the balloon with antiproliferative drugs, thereby reducing the risk of in-stent restenosis after PCI and the formation of late stent thrombosis (2, 3). Adequate pretreatment of the target vessel is key to the efficacy of DCB treatment (4–6). Physiological indicators, such as the fractional flow reserve (FFR) and instantaneous wave-free ratio, can effectively guide vascular pretreatment in interventional therapy. Notably, FFR-guided coronary interventions result in a better prognosis than coronary angiography (7).

Although the FFR is widely considered the gold standard for assessing coronary vessel physiology, its application in clinical practice is not universal, perhaps owing to costly pressure guidewires, additional procedural steps, an increased procedure time, and the side effects of adenosine drugs. In contrast, the quantitative flow ratio (QFR) is based on the physiological characteristics of the coronary artery and can be used to assess functional coronary stenosis without using a pressure guidewire. The QFR is simple, rapid, economical, and suitable for assessing coronary stenosis in various clinical settings. Moreover, the QFR outperforms the FFR in terms of specificity and sensitivity (8–10). Based on previous functional studies, the 2020 global DCB expert consensus (11) recommends using the FFR to better evaluate coronary function in cases of dissection and severe residual stenosis after acute lumen acquisition, with a threshold of 0.80 to achieve a better prognosis. However, studies reporting on pretreating target lesions under QFR guidance are scarce. Therefore, this study investigated the predictive value of QFR-guided preconditioning of target vessels for functional stenosis at the mid-term follow-up in patients treated with DCB.

## 2 Methods

### 2.1 Study design and population

This single-center, retrospective, observational cohort study included adults aged ≥18 years with CAD who received pharmacological balloon therapy between August 2021 and December 2023 at the Second Affiliated Hospital of Fujian Medical University. Our large regional medical center routinely employs drug-coated balloon (DCB) angioplasty as a conventional treatment strategy. All operators in this study were proficient in this technique. All included patients had complete follow-up data and coronary angiography (CAG) images, and a QFR analysis was performed using those CAG images. We excluded patients with severe vessel tortuosity, vessel overlap, CAG image sharpness that did not support the QFR analysis, severe aneurysmal dilatation of the target vessel, combined tumors, severe renal insufficiency (creatinine >150 mmol/L or estimated glomerular filtration rate <45 ml/kg/1.73 m^2^), previous coronary artery bypass grafting, and completely occluded target vessels. Coronary stenosis is generally considered functionally significant when the QFR values are ≤0.80. Thus, the patients were placed in high (QFR >0.80) or low (QFR ≤0.80) groups based on the QFR value at follow-up.

The Ethics Committee of the Second Affiliated Hospital of Fujian Medical University approved this study. Each patient provided consent for participation in this retrospective observational cohort study.

### 2.2 Target vessel treatment

After angiography, the appropriate guide catheter and working guide wire were selected based on the culprit vessel. The guide wire was controlled through the culprit lesion of the target vessel to the distal segment of the vessel. Conventional or special (e.g., non-compliant or cut) balloons were selected for adequate pre-dilation (balloon-to-blood vessel diameter ratio: 0.8–1.0:1). Preconditioning was performed in 10 s cycles at 6–14 atm for one to several cycles based on the vasodilatation. Then, nitroglycerin (100 or 200 µg) was administered in the coronary artery. Angiography was used to determine the preconditioning effect and whether the target vessel simultaneously met the following conditions: (1) thrombolysis in myocardial infarction blood flow grade III; (2) no type C or above dissection; and (3) a visual diameter stenosis rate of <30%. Target vessels meeting these conditions were considered adequately pretreated and to have met the vascular conditions for DCB treatment. DCB treatment was delivered to the target lesion; the pressure was expanded for 60–120 s. The effects of the DCB treatment were determined by angiography; then, the target vessel was observed for 10 min to detect whether it had elastic recoil and blood flow. Rescue DES implantation was considered if obvious elastic recoil, severe dissection, or slow coronary flow was observed; in these instances, the case was excluded from this study. In addition, the intravascular ultrasound (IVUS) utilization rate, influenced by strict national health insurance reimbursement policies and patient economic factors, served to minimize its potential confounding effect on the study's primary endpoint.

### 2.3 Clinical data sources and QFR analyses

We retrospectively collected data on patient clinical features, laboratory tests, vascular anatomical features, interventional procedure data, and QFR data from the National Health System. Based on the CAG images, a QFR analysis of the best single view of the target vessel was performed by two cardiology interventionalists qualified in interventional therapy and trained in computational coronary physiology using the QFR Measure System 3.0. The two interventionalists who performed the off-line QFR analysis were blinded to all clinical and procedural data. The QFR values of the target vessels were measured at baseline, after pretreatment, upon DCB treatment, and at the mid-term follow-up using the QFR measurement system to calculate the target vessel parameters.

### 2.4 Postprocedural medication

All patients received dual antiplatelet therapy (DAPT) with aspirin and a P2Y12 receptor inhibitor for 1–3 months. In those with high bleeding risk, DAPT duration was shortened as clinically indicated; otherwise, DAPT was de-escalated to single antiplatelet therapy with either aspirin or clopidogrel after 3 months. For patients presenting with acute coronary syndrome (ACS), a minimum 12-month DAPT regimen was mandated. Statin therapy was initiated in all cases, with lipid-lowering strategies individually titrated based on serial outpatient lipid profile monitoring. Additionally, guideline-directed medical therapy was optimized, including angiotensin-converting enzyme inhibitors/angiotensin II receptor blocker/angiotensin receptor-neprilysin inhibitors (ACEI/ARB/ARNI), *β*-blockers, and ezetimibe as appropriate.

### 2.5 Statistical analyses

Normally distributed continuous variables were compared using Student's *t*-test and presented as means ± standard deviations. Discrete continuous variables were compared using the Mann–Whitney *U* test and presented as medians. Categorical variables were compared using chi-square or Fisher's exact tests and presented as *n* (%). We used a univariate logistics regression analysis to investigate the association of each possible independent predictor of functional stenosis at the mid-term follow-up for patients with coronary heart disease treated with DCB. Variables with *P*-values of <0.20 were included in the multiple logistics regression analysis, and adjusted odds ratios (ORs) and 95% confidence intervals (CIs) were calculated. A receiver operating characteristic (ROC) curve analysis and Youden's index were used to determine the QFR cutoff values related to QFR >0.8 in the overall study group and the pretreated small vessel subgroup. The predictive ability was evaluated based on the area under the curve (AUC). Kaplan–Meier survival curves were used to visualize freedom from functional stenosis during follow-up in pretreatment QFR groups, with group differences compared by the Log-rank test. To explore the possible mediating effect of the QFR after pretreatment and DCB treatment on the outcome, the mediating effect was tested using SPSS Model 4. The bootstrap method by Hayes was used to verify if the QFR after DCB treatment elicited a mediating effect between the QFR after pretreatment and the QFR at follow-up (https://www.processmacro.org). The small-vessel subgroup (diameter ≤2.75 mm) was analyzed simultaneously. *P*-values of <0.05 were considered statistically significant. All statistical analyses were performed using R version 4.4.1 (R Core Team, Vienna, Austria) and IBM SPSS Statistics for Windows version 27.0 (IBM Corp., Armonk, NY, USA).

## 3 Results

### 3.1 Baseline characteristics

**Figure 1:**
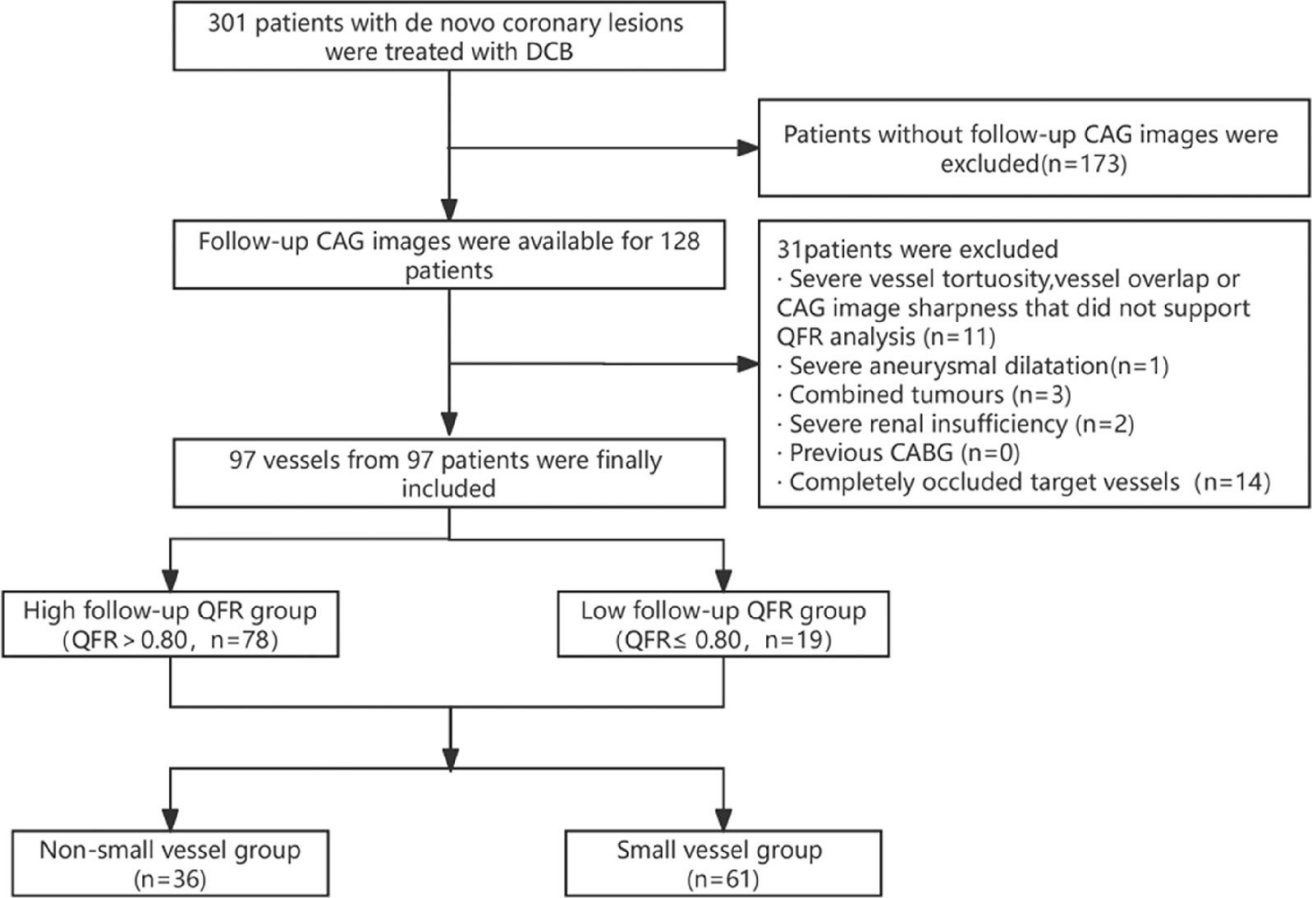
Study flowchart. The study included 97 vessels from 97 patients that met the strict inclusion and exclusion criteria. Patients were divided into high (QFR >0.8) and low (QFR ≤0.8) QFR groups based on the QFR at the mid-term follow-up. Baseline analyses focused on the QFR groups and the small vessel group. CABG, coronary artery bypass grafting; CAG, coronary angiography; DCB, drug-coated balloon; QFR, quantitative flow ratio.

**Figure 2:**
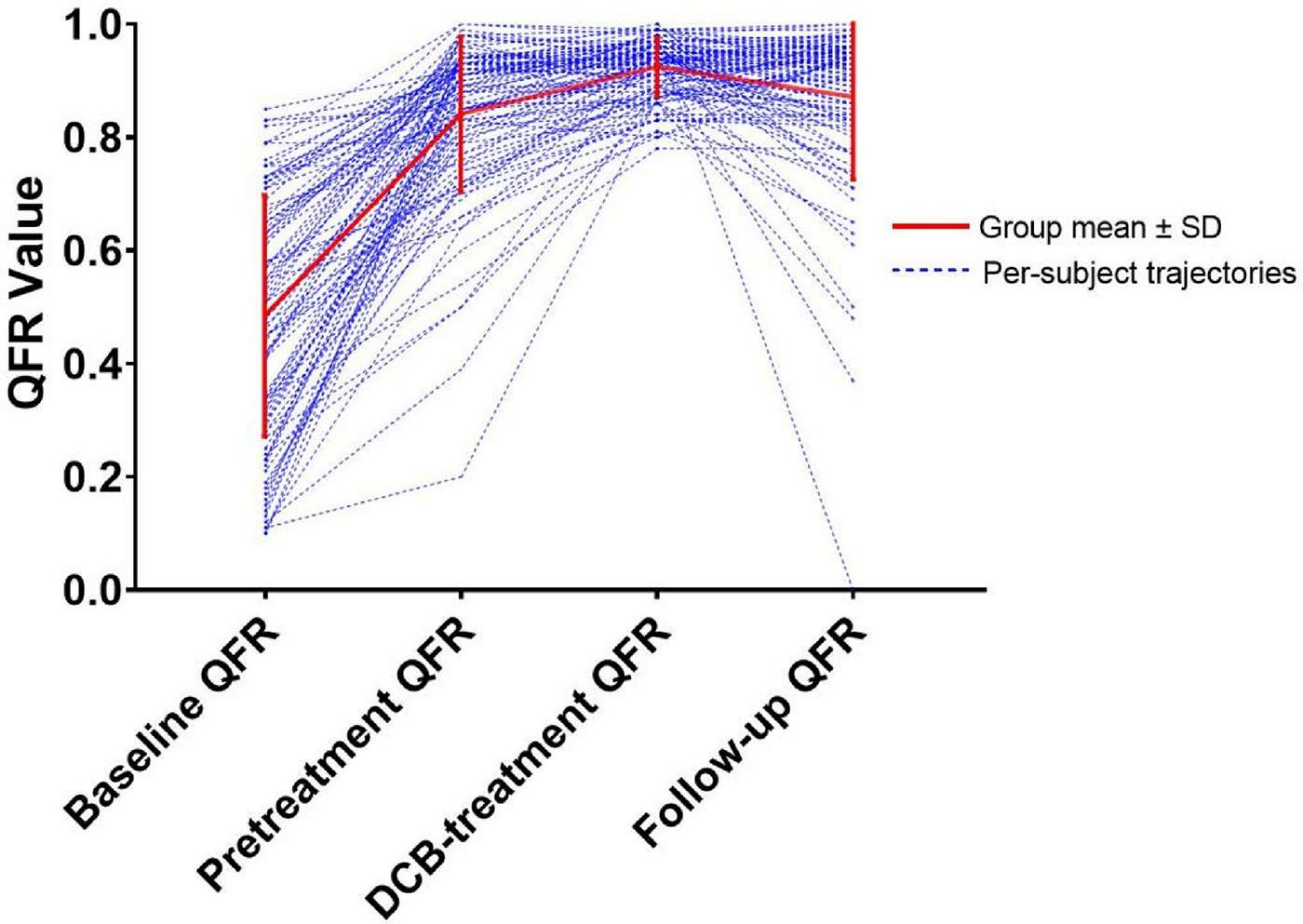
A line graph depicting the evolution of QFR values at four critical time points. QFR, quantitative flow ratio. DCB, drug-coated balloon.

Initially, 301 patients were enrolled, of which 114 of 115 *de novo* coronary lesions underwent primary PCI and follow-up CAG. After applying the exclusion criteria, the study included 97 vessels from 97 patients (Figure 1). The median follow-up time was 297.0 days, and the mean age was 58.20 ± 11.02 years. Furthermore, 24 patients (24.74%) had unstable angina pectoris, 16 (16.50%) had acute non-ST-segment elevation myocardial infarction, and 57 (58.76%) had stable angina pectoris. The mean baseline QFR value was 0.48 ± 0.21, which increased to 0.84 ± 0.14 after sufficient pretreatment and further increased to 0.92 ± 0.05 after DCB treatment. The mean follow-up QFR was 0.87 ± 0.15 (Figure 2). The high (QFR >0.80) and low (QFR ≤0.80) QFR groups included 78 (80.4%) and 19 (19.6%) vessels, respectively. Of the clinical features, laboratory tests, vascular anatomical features, and interventional procedure data, only the glycosylated hemoglobin level significantly differed between the two groups. Pretreatment QFR showed statistically significant differences between functional and non-functional stenosis groups (*P* = 0.013), while baseline QFR and DCB-treatment QFR showed no statistically significant differences between the two groups (Table 1).

**Table 1:** Patients were divided into two groups according to follow-up QFR.

Per-patient analysis	Total (*n* = 97)	Low (*n* = 19)	High (*n* = 78)	*P* value
Clinical features				
Age (years)	58.20 ± 11.02	55.44 ± 11.70	58.88 ± 10.83	0.239
Female	18 (18.56)	2 (10.53)	16 (20.51)	0.500
Hypertension	54 (55.67)	10 (52.63)	44 (56.41)	0.766
Smoking	30 (30.93)	6 (31.58)	24 (30.77)	0.945
Hyperlipidemia	41 (42.27)	9 (47.37)	32 (41.03)	0.616
Diabetes	34 (35.05)	4 (21.05)	30 (38.46)	0.154
Previous MI	3 (3.09)	1 (5.26)	2 (2.56)	0.484
Previous PCI	13 (13.40)	1 (5.26)	12 (15.38)	0.432
COPD	10 (10.31)	3 (15.79)	7 (8.97)	0.649
PAD	2 (2.06)	0 (0.00)	2 (2.56)	1.000
BMI (kg/m^2^)	24.32 ± 2.97	24.68 ± 2.80	24.24 ± 3.02	0.562
Drinking history	16 (16.49)	4 (21.05)	12 (15.38)	0.801
Clinical diagnosis	40 (41.24)	9 (47.37)	31 (39.74)	0.726
Stable angina pectoris	57 (58.76)	10 (52.63)	47 (60.25)	
Unstable angina pectoris	24 (24.74)	6 (31.58)	18 (23.08)	
NSTEMI	16 (16.50)	3 (15.79)	13 (16.67)	
Laboratory tests				
LVEF (%)	59.54 ± 8.63	59.00 ± 11.93	59.67 ± 7.71	0.819
HB (g/L)	141.12 ± 15.86	140.84 ± 12.85	141.19 ± 16.58	0.932
LDL-C (mmol/L)	3.03 ± 1.07	2.96 ± 0.89	3.04 ± 1.12	0.778
TG (mmol/L)	2.01 ± 1.84	2.26 ± 2.54	1.95 ± 1.64	0.514
TC (mmol/L)	4.82 ± 1.31	4.72 ± 0.97	4.84 ± 1.38	0.731
Scr (umol/L)	81.23 ± 29.07	83.15 ± 16.19	80.76 ± 31.48	0.750
HbAlc (%)	6.51 ± 1.41	5.87 ± 0.79	6.67 ± 1.49	0.027
NT-ProBNP (pg/ml)	162.00 (60.89, 618.00)	89.00 (46.83, 505.10)	205.55 (68.66, 798.83)	0.106
hs-cTnT (ng/L)	13.91 (7.38, 200.30)	32.74 (8.79, 854.80)	12.45 (6.63, 184.40)	0.148
Vascular anatomical features				
Small vessel	61 (62.89)	12 (63.16)	49 (62.82)	0.978
Angiographic disease severity				0.566
1-vessel disease	56 (57.73)	10 (52.63)	46 (58.97)	
2-vessel disease	20 (20.62)	3 (15.79)	17 (21.79)	
3-vessel disease	21 (21.65)	6 (31.58)	15 (19.23)	
Culprit vessel				0.603
LAD	47 (48.45)	10 (52.63)	37 (47.44)	
LCX	32 (32.99)	7 (36.84)	25 (32.05)	
RCA	18 (18.56)	2 (10.53)	16 (20.51)	
DS (%)	60.27 ± 12.06	61.17 ± 11.07	60.06 ± 12.35	0.719
AS (%)	82.75 ± 10.96	83.77 ± 10.03	82.50 ± 11.22	0.652
Reference luminal diameter (mm)	2.60 ± 0.57	2.61 ± 0.62	2.59 ± 0.57	0.913
Length of lesion (mm)	26.21 ± 13.82	29.75 ± 19.52	25.35 ± 12.05	0.215
LM	2 (2.06)	0 (0.00)	2 (2.56)	1.000
IVUS	11 (11.34)	3 (15.79)	8 (10.26)	0.781
Interventional procedure data				
DCB diameter (mm)	2.87 ± 0.44	2.70 ± 0.44	2.91 ± 0.43	0.062
DCB length (mm)	25.60 ± 7.41	25.32 ± 6.73	25.67 ± 7.60	0.854
DCB pressure (atm)	9.00 ± 1.04	8.84 ± 1.01	9.04 ± 1.05	0.464
DCB expansion time (s)	63.09 ± 15.90	60.00 ± 0.00	63.85 ± 17.67	0.347
Pretreatment balloon diameter (mm)	2.73 ± 0.43	2.59 ± 0.44	2.76 ± 0.42	0.119
Pretreatment balloon length (mm)	15.13 ± 2.09	15.00 ± 2.36	15.17 ± 2.04	0.758
Pretreatment balloon pressure (atm)	10.23 ± 1.78	9.79 ± 1.75	10.33 ± 1.78	0.233
Pretreatment expansion time (s)	18.66 ± 7.72	19.47 ± 8.48	18.46 ± 7.57	0.611
Cutting balloon	57 (58.76)	11 (57.89)	46 (58.97)	0.932
Coronary dissection	3 (3.09)	0 (0.00)	3 (3.85)	1.000
QFR				
Baseline QFR	0.48 ± 0.21	0.49 ± 0.23	0.48 ± 0.21	0.898
Pretreatment QFR	0.84 ± 0.14	0.77 ± 0.17	0.85 ± 0.12	0.013
DCB-treatment QFR	0.92 ± 0.05	0.91 ± 0.06	0.92 ± 0.05	0.446
Follow-up QFR	0.87 ± 0.15	0.64 ± 0.19	0.92 ± 0.05	<.001

Data are presented as mean ± SD, median (IQR), or *n* (%).

QFR, quantitative flow ratio; MI, myocardial infarction; PCI, percutaneous coronary intervention; COPD, chronic obstructive pulmonary disease; PAD, peripheral arterial disease; BMI, body mass index; NSTEMI, non-ST-segment elevation myocardial infarction; LVEF, left ventricular ejection fraction; HB, hemoglobin; LDL-C, low-density lipoprotein cholesterol; TG, triglyceride; TC, total cholesterol; Scr, serum creatinine; NT-ProBNP, N-terminal pro-brain natriuretic peptide; hs-cTnT, high sensitivity cardiac troponin T; LAD, left anterior descending; LCX, left circumflex; RCA, right coronary artery; DS, diameter stenosis; AS, area stenosis; LM, left main disease; IVUS, intravascular ultrasound; DCB, drug-coated balloon.

### 3.2 Risk factors for functional stenosis at follow-up

The univariate logistics regression analysis identified diabetes mellitus, N-terminal prohormone of brain natriuretic peptide, glycosylated hemoglobin, the QFR after pretreatment, the pretreatment balloon diameter, and the drug balloon diameter as significant factors. (Of note, since the pretreatment and drug balloon diameters are similar in clinical practice, we included only the balloon diameter after preconditioning to avoid collinearity.). The multivariate logistic regression equation was constructed with these variables, and the results showed that the higher the QFR after pretreatment, the lower the risk of functional stenosis at follow-up (OR: 0.96, 95% CI: 0.92–0.99, *P* = 0.041) (Table 2).

**Table 2:** Univariate and multivariate logistics regression analyses for predicting low QFR at follow-up.

Variables	Univariate analysis		Multivariable Analysis	
	OR (95% CI)	*P* value	Adjusted OR (95% CI)	*P* value
Pretreatment QFR, per 0.01 unit	0.96 (0.93–0.99)	0.022	0.96 (0.92–0.99)	0.041
Diabetes	0.43 (0.13–1.41)	0.162	2.47 (0.35–17.33)	0.364
Pretreatment balloon diameter	0.37 (0.11–1.30)	0.122	0.60 (0.15–2.44)	0.478
NT-ProBNP	1.00 (1.00–1.00)	0.190	1.00 (1.00–1.00)	0.260
HbAlc	0.51 (0.27–0.98)	0.045	0.38 (0.13–1.11)	0.077

CI, confidence interval; HbA1c, hemoglobin A1C; NT-ProBNP, N-terminal prohormone of brain natriuretic peptide; OR, odds ratio; QFR, quantitative flow ratio.

### 3.3 ROC curve analysis

**Figure 3:**
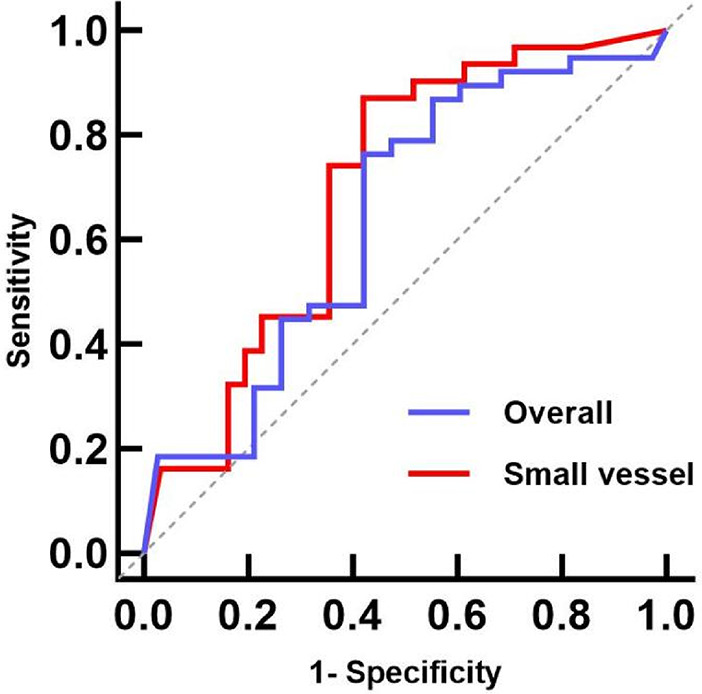
Pretreatment QFR ROC curves for predicting the mid-term follow-up QFR. The AUCs for the overall and small vessel groups were 0.638 (95% CI: 0.478–0.789, *P* = 0.051) and 0.723 (95% CI: 0.536–0.909, *P* = 0.016), respectively. ROC, receiver operating characteristic; QFR, quantitative flow ratio; AUC, area under the curve.

The ROC curve analysis of the overall group identified a pretreatment QFR cutoff value of 0.705 for predicting the functional stenosis outcome at follow-up (AUC: 0.643, 95% CI: 0.501–0.785; sensitivity: 0.350; specificity: 0.935; *P* = 0.049). The cutoff value for the small vessel group was also 0.705 (AUC: 0.723, 95% CI: 0.536–0.909; sensitivity: 0.500; specificity: 0.939; *P* = 0.016). These results suggest that a QFR >0.705 after pretreatment can predict whether the target vessel will have functional stenosis at the mid-term follow-up, especially in small vessels (Figure 3).

### 3.4 Post hoc power analysis

To assess whether the current sample size had sufficient statistical power, a *post hoc* power analysis was performed using the ClinCalc online calculator (https://clincalc.com/stats/power.aspx). This study was concerned with whether the functional stenosis correlation analysis was statistically powered. Relevant data listed in Table 3 were entered into the online calculator (i.e., p1 = 53.85%, n1 = 13, p2 = 14.29%, n2 = 84), and the statistical power was 87.3%, which was above the ideal threshold (ideal power = 80%). The results showed that the effect size of the proportion difference between groups was large, the overall sample size was sufficient, and the correlation analysis of functional stenosis after pretreatment was statistically powerful. This process was repeated for the small vessel subgroup (Table 3), and the *post hoc* statistical power in small vessels was 90.7%. This value was also greater than the ideal threshold and held statistical power. The Cohen's h values were calculated as 0.87 for the full sample and 1.07 for the small-vessel subgroup, both of which exceed the conventional threshold for a large effect size (*h* > 0.8). The effect size observed between groups suggests that the analysis retains a reasonable degree of statistical power.

**Table 3:** Incidence of functional stenosis in the overall and small vessel groups.

Category	Total, *N*	Functional stenosis, % (*n*/*N*)	Low pretreatment QFR group (QFR ≤ 0.705), % (*n*/*N*)	High pretreatment QFR group (QFR > 0.705), % (*n*/*N*)	*P* value
Overall	97	19.59 (19/97)	53.85 (7/13)	14.29 (12/84)	0.003
Small Vessel	61	19.67 (12/61)	60.00 (6/10)	11.76 (6/51)	0.002

QFR, quantitative flow ratio.

### 3.5 Kaplan–Meier analysis for functional stenosis

**Figure 4:**
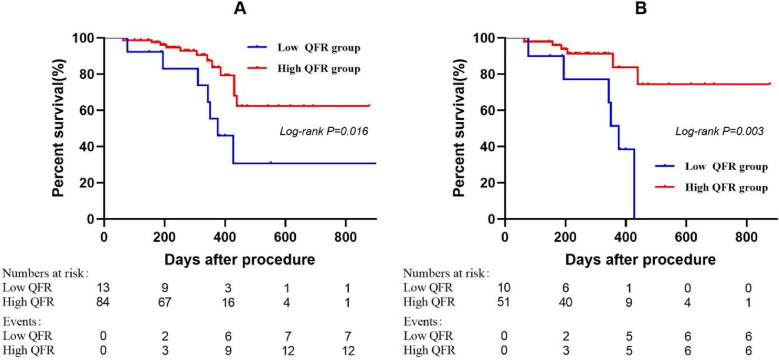
Kaplan–meier curves showing probability of survival stratified by the pretreatment QFR. The groups were stratified by the optimal cutoff value of the pretreatment QFR determined by receiver operating characteristic curve analysis. **(A)** Entire cohort. **(B)** Small vessel subgroup. QFR, quantitative flow ratio.

Laboratory tests and concomitant medications during follow-up are presented in Table 4, and no statistically significant differences were observed between groups (all *P* > 0.05). Kaplan–Meier analysis demonstrated a significant association between dichotomized pretreatment QFR (<0.705 vs. ≥0.705) and subsequent functional stenosis (Log-rank *P* = 0.016; Figure 4A). The low pretreatment QFR group exhibited significantly higher incidence of functional stenosis compared to the high QFR group (53.85% vs. 14.29%; *P* = 0.003; Table 3). In the small vessel subgroup, pretreatment QFR stratification similarly predicted functional stenosis (Log-rank *P* = 0.003; Figure 4B). The incidence disparity was more pronounced (60.00% vs. 11.76%; *P* = 0.002; Table 3).

**Table 4:** Patients were divided into two groups according to follow-up QFR.

Per-patient analysis	Total (*n* = 97)	Low (*n* = 19)	High (*n* = 78)	*P* value
Laboratory tests				
HB (g/L)	139.84 ± 16.88	139.32 ± 13.29	139.96 ± 17.72	0.882
LVEF (%)	60.25 ± 8.78	60.79 ± 8.54	60.12 ± 8.89	0.766
Scr (umol/L)	86.16 ± 32.73	89.35 ± 23.99	85.39 ± 34.62	0.638
LDL-C (mmol/L)	1.79 ± 0.64	1.80 ± 0.70	1.79 ± 0.63	0.945
TG (mmol/L)	1.65 ± 1.13	1.67 ± 1.28	1.64 ± 1.10	0.926
TC (mmol/L)	3.58 ± 0.92	3.61 ± 0.90	3.57 ± 0.93	0.860
HbAlc (%)	6.19 ± 0.56	6.08 ± 0.37	6.21 ± 0.60	0.356
Additional medication				
Beta-blocker	75 (77.32)	15 (78.95)	60 (76.92)	1.000
ACEI/ARB/ARNI	62 (63.92)	9 (47.37)	53 (67.95)	0.094
Ezetimibe	12 (12.37)	3 (15.79)	9 (11.54)	0.908

All patients received dual antiplatelet therapy and statin treatment. Data are presented as mean ± SD or *n* (%).

QFR, quantitative flow ratio; HB, hemoglobin; LVEF, left ventricular ejection fraction; Scr, serum creatinine; LDL-C, low-density lipoprotein cholesterol; TG, triglyceride; TC, total cholesterol; ACEI, angiotensin-converting enzyme inhibitors; ARB, angiotensin II receptor blocker; ARNI, angiotensin receptor-neprilysin inhibitors.

### 3.6 Mediation effect analysis

**Figure 5:**
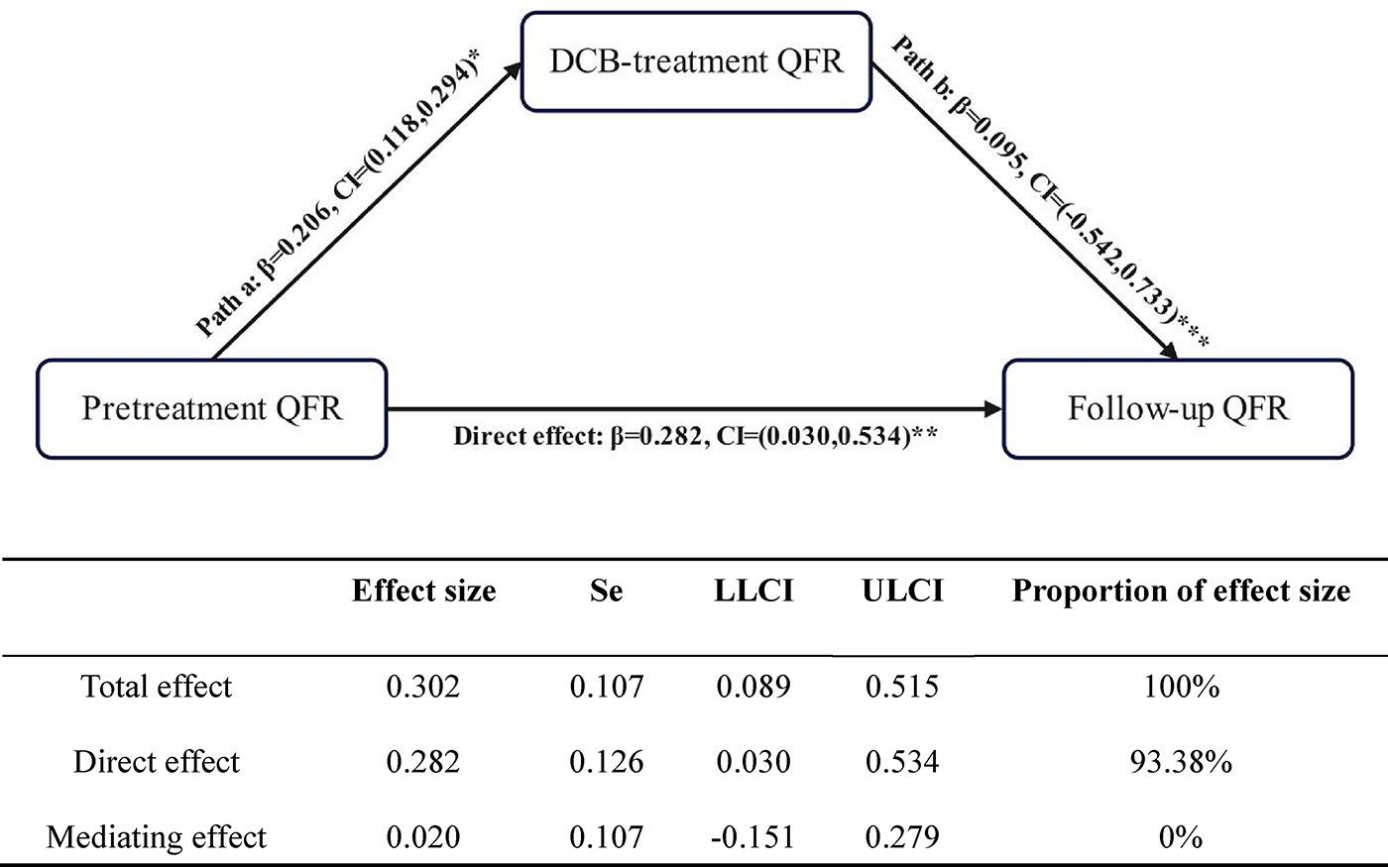
Meditating effect of the DCB-treatment QFR on the pretreatment and follow-up QFR. *P < 0.001; **P = 0.029; ***P = 0.766. DCB, drug-coated balloon; LLCI, lower limit confidence interval; QFR, quantitative flow ratio; Se, standard error; ULCI, upper limit confidence interval.

The bootstrap method (Model 4) was used to perform a simple mediating effect analysis using the pretreatment QFR as the independent variable (X), the follow-up QFR as the dependent variable (Y), and the DCB-treatment QFR as the mediating variable (M). The upper and lower 95% CIs (ULCI and LLCI, respectively) of the effect of the pretreatment QFR on the follow-up QFR (*β* = 0.282, LLCI = 0.030, ULCI = 0.534) and DCB-treatment QFR (*β* = 0.206, LLCI = 0.118, ULCI = 0.294) did not include 0. In contrast, the upper and lower limits of the bootstrap 95% CIs of the mediating effect included 0 (*β* = 0.020, LLCI = −0.151, ULCI = 0.279), indicating that pretreatment QFR directly affected the follow-up QFR. However, the mediating effect of the DCB-treatment QFR was not significant. The direct effect of the pretreatment QFR (0.282) accounted for 93.38% of the total effect (0.302) (Figure 5).

## 4 Discussion

This study's main findings were: (1) target vessels with a low pretreatment QFR had a higher risk of functional stenosis at follow-up; (2) the cutoff value for predicting this outcome was 0.705; and (3) in small vessels, the pretreatment QFR directly influenced the follow-up QFR.

The pretreatment phase, especially the outcome assessment after pre-expansion, is crucial for the subsequent treatment strategy. Evaluations can be based on angiographic and physiological parameters (11, 12) that reveal blood vessel characteristics from different perspectives and may influence treatment decisions. A previous study reported mismatches between angiographic and physiological indicators in >75% of cases (7). The current consensus is that drug balloon implantation can be considered when the vessel's anatomical stenosis is <30%; however, the percent diameter stenosis (DS%) provides limited insight into the anatomical characteristics of the vessel at the vessel's narrowest part. Furthermore, this measurement may be affected by the operator's subjective judgment, thus deviating from the DS% calculated objectively by artificial intelligence technology. Physiological assessments can reveal hemodynamic abnormalities that cannot be visually identified by angiography (13). In the present study, although the mean stenosis rate of the small vessels was 27.34%, more than one-third of the vessels (26 of 61) had a stenosis rate >30%. Therefore, performing surgery according to the guideline standards may deviate from the actual condition. In addition, strict adherence to the guidelines' principles may disqualify some patients from DCB therapy. A large study reported that even if treated with cutting or scoring balloons, 21% of patients still do not meet the criteria for pre-dilation success and instead receive implanted stents (14). In the case of complex lesions, such as diffuse long lesions and bifurcation lesions, choosing DES instead of DCB will greatly increase the difficulty and duration of the operation, thereby increasing the associated surgical risk and the risk of long-term in-stent restenosis. Therefore, using physiology to evaluate the pretreatment is critical, yet clear guidance and related studies on appropriate pre-expansion strategies based on QFR guidance are lacking.

Previous studies have shown that drug penetration in the intimal and medial layers of elastic arteries is limited under normal and atherosclerotic vascular conditions, and the intact arterial wall poses a significant barrier to drug penetration (15, 16). Sufficient pre-dilation of blood vessels by pretreatment damages the intima and media of blood vessels and creates favorable conditions for DCB penetration. After endothelial injury, drugs can quickly penetrate the vascular wall and effectively inhibit the proliferation and migration of smooth muscle cells, which is crucial for preventing vascular restenosis. Notably, the PICCOLETTO study (17, 18) was terminated early because of the high rate of major adverse cardiovascular events in the DCB group due to inadequate preparation for balloon pre-dilatation, emphasizing the importance of adequate pretreatment, which our study confirmed.

Small vessel diameter is the most influential factor among many clinical variables in the development of restenosis (19). Thus, the small vessel treatment strategy should focus on effective restenosis control (20). Jeger et al. (21) demonstrated the safety of DCB as a treatment option for small-vessel CAD after successful pre-dilation, provided that the angiographic results met the acceptable criteria. Under these conditions, DCB treatment belongs to an “interventional without implantation” approach, which reduces the intravascular inflammatory response caused by the implant and, thus, the risk of restenosis. Several clinical randomized controlled trials have demonstrated that DCB treatment is safe and feasible and significantly reduces the risk of small vessel disease (SVD) restenosis. They have also confirmed that DCB is not inferior to DES implantation in terms of clinical efficacy (21–24). A recent open-label, randomized, non-inferiority trial conducted in 43 centers in China reaffirmed the non-inferior long-term efficacy of DCB compared with DES for treating SVD (14). Unfortunately, the incidence of restenosis of small vessels is still high after reasonable treatment (25). Therefore, our study focused on the small-vessel subgroup, finding that some results were more statistically significant than those in the overall group.

Our study suggests a low pretreatment QFR may promote functional stenosis at follow-up. The FAME-2 study (26) confirmed that for patients with coronary artery lesions with physiological ischemia, PCI plus medical therapy significantly reduced the rate of emergency revascularization and recurrent angina compared with medical therapy alone. Although asymptomatic, functional stenosis predicts a significant increase in future cardiovascular risk events in patients who undergo planned angiography after DCB treatment. Active revascularization may improve the prognosis of such patients and provide continued benefit. In this study, a pretreatment QFR >0.705 was a protective factor for functional stenosis at follow-up in the overall group and the small-vessel subgroup. Notably, although this value is far from the physiological target value recommended by the consensus (FFR >0.80), we suspect that rigorous conditioning is more likely to cause excessive damage to the fragile small vessels, which may cause complications and increase the risk of future functional stenosis. Some patients receive stents because of dissection or hematoma that affects blood flow. Thus, striking a balance of “enough but not too much” during conditioning may be a key strategy to reduce the risk of complications, maintain vascular patency, and create favorable conditions for subsequent therapy.

To our knowledge, the present study is the first to focus on the interrelationship among QFR values after pretreatment, after DCB release, and at follow-up. Although the DCB-treatment QFR was significantly associated with the pretreatment QFR, the pretreatment QFR did not affect the follow-up QFR via the DCB-treatment QFR. Instead, the pretreatment QFR directly affected the follow-up QFR. The mediating effect analysis results suggest that, first, pretreatment mainly involves enlarging the lumen to improve hemodynamics, and this benefit may positively correlate with functional indicators immediately after DCB. Second, although DCB has a certain vasodilator effect, the balloon is only the carrier of drug delivery, and the drugs' permeability is the key factor of DCB treatment. Third, although the target vessel's QFR may improve after DCB treatment compared to after pretreatment, pretreatment is the main factor affecting the prognosis. The prognosis may be poor in cases of insufficient pretreatment, even if a satisfactory QFR after DCB treatment is obtained. This suggests we should pay more attention to adequate but not excessive pretreatment of vascular target lesions.

Our study had some limitations. First, this is a single-center retrospective clinical study with selection bias. Second, we prespecified ischemia-driven target vessel revascularization (TVR) as a clinical outcome for the study. However, we noted that patients with ischemic symptoms who had to return to the hospital for repeat angiography with TVR were recorded as having a positive outcome. Some patients declined further TVR, and some asymptomatic patients were not enrolled because they did not return for repeat angiography. As a retrospective study, these factors have a large bias. Although the focus on the functional outcome in this study potentially reduced this bias, and the functional outcome can predict a poor prognosis, the existing clinical events are still of interest. For our study, the TVR rate was 10.3% (10 cases) in the overall cohort, but in the low QFR group, the proportion of TVR increased to nearly half (9 cases, 47.3%), highlighting the need for future large-scale studies to further elucidate the relationship between low QFR and adverse clinical outcomes. Third, our results are limited to this study population and require confirmation in prospective, multicenter, randomized controlled studies. Fourth, we could not conduct sufficient statistical analysis on the subgroups of complex lesions or different lesion vessels and locations due to the sample size limitation of a single-center study. These differences may affect the results; thus, a larger sample size should be included in further analyses.

## 5 Conclusions

We found that in target vessel disease, especially in SVD, the QFR after pretreatment significantly and directly affected the follow-up QFR. Moreover, QFR values <0.705 could be a valuable predictor of functional stenosis after DCB treatment, and QFR values of 0.903–0.930 after DCB treatment were associated with good functional outcomes at follow-up. These results provide new guidance for treating CAD.

## Data Availability

The data analyzed in this study is subject to the following licenses/restrictions: Requires the approval of the relevant department of the organization. Requests to access these datasets should be directed to the corresponding author.
